# Initiation of rest-grazing during soil thawing improves interspecific relationships and stability of plant communities on alpine grassland

**DOI:** 10.3389/fpls.2024.1426626

**Published:** 2024-08-06

**Authors:** Kaikai Ma, Qingqing Hou, Changlin Xu, Yanzhu Chen, Hang Yang, Jingjing Xu, Xiaojun Yu

**Affiliations:** ^1^ College of Grassland Science, Key Laboratory of Grassland Ecosystem (Ministry of Education), Gansu Agricultural University, Lanzhou, China; ^2^ Department of Forestry Administration of Gansu Province, Gansu Forestry Technology Promotion Station, Lanzhou, China

**Keywords:** alpine meadows, rest-grazing, niche, interspecific relationship, community stability

## Abstract

**Introduction:**

Grazing management is essential to maintain the stability of grassland ecosystems.

**Methods:**

To determine the optimal rest-grazing period of alpine meadow, five rest-grazing periods were set based on soil thawing and plant re-greening in this study. The niche, interspecific relationships, and stability of plant communities at different rest-grazing periods were investigated.

**Results:**

Rest-grazing during soil thawing resulted in a small niche width and niche overlap of plants, overall positive interspecific associations, and a high stability of plant communities. Delayed rest-grazing time to plant re-greening resulted in a large niche width and niche overlap of plants, overall negative interspecific associations, and a low stability of plant communities.

**Discussion:**

Rest-grazing in alpine meadows should begin as soon as possible to promote healthy and sustainable utilization of grasslands.

## 1 Introduction

Alpine meadows are the largest grassland types on the Qinghai-Tibet Plateau (QTP) (Niu et al., 2019), and are crucial to protect biodiversity, maintain stability of regional ecosystems, and promote the development of animal husbandry (Liu et al., 2012). However, due to the influence of climate change and human activities, alpine meadows have shown varying degrees of degradation in recent years, not only affecting animal production and herding livelihoods, but also threatening the ecological security of the country (Miehe et al., 2019). Studies have shown that overgrazing is the primary cause of the degradation of alpine grassland ecosystems on the QTP (Kemp et al., 2013). Overgrazing can considerably reduce plant density and biomass (Wang et al., 2019a), thus affecting plant niches and interspecific relationships (Wu et al., 2023). Grazing management is crucial for maintaining both the biodiversity and stability of alpine grassland ecosystems.

The classical niche theory holds that niche differentiation among species is caused by differences in resource utilization, which is one of the basic mechanisms of species coexistence and the main driving force of community structure change and vegetation succession (Wei et al., 2015). Grazing disturbance is an important factor causing differences in resource utilization. Grazing inhibits the growth of palatable plants, promotes the growth and development of other plants, alters the utilization of resources by plant communities, and affects species niche width, niche overlap, and interspecific relationships (Liu et al., 2019; Pulungan et al., 2019). Currently, research on the effects of grazing management on niche and interspecific relationships has focused on grazing intensity and enclosure, while those on niche and interspecific relationships among plants in alpine meadows during the rest-grazing period remain limited.

Reasonable rest-grazing is an important measure of grassland management, and has become one of the effective measures for ecological restoration of degraded grassland (Zimmer et al., 2010; Li et al., 2017). Most existing studies suggest that the rest-grazing of cold season pastures in alpine meadows starts at the plant re-greening stage. However, our previous observations have shown that surface soil thawing and freezing occur before plant re-greening. Trampling by grazing livestock during this period peels the thawed turf from grasslands or forms a “hoof pit”, which damages underground plant organs and negatively affects plant re-greening (Pan et al., 2021; Peng et al., 2022). Therefore, five rest-grazing periods were set based on the depth of soil thawing and the coverage of plant re-greening to determine the effects of rest-grazing time on species niche, interspecific relationship and stability of plant communities in alpine meadows. We proposed three hypotheses: (1) the maximum niche overlap of plants will occur between two species with larger niche width; (2) interspecific relationships will positively correlate with stability of plant communities; and (3) early rest-grazing will improve interspecific relationships and stability of plant communities.

## 2 Materials and methods

### 2.1 Experimental site

The study site is located in the Tianzhu alpine meadow on the northeastern edge of the QTP in the Jinqiang River Valley (37°40′N, 102°32′E) at an elevation of 2960 m. The annual average temperature of the study site is −0.1°C, the average annual accumulated temperature is 1380°C, annual precipitation is 416 mm and annual evaporation reaches 1592 mm. The study area has no absolute frost-free period, and the seasons are only divided into cold and hot seasons. *Carex capillifolia* is the dominant plant species, and *Polygonum viviparum* is the constructive plant species. The accompanying plant species include *Carex alatauensis*, *Helictotrichon tibeticum*, *Artemisia smithii*, and *Medicago ruthenica* var. *inschanica*.

### 2.2 Experimental design

A typical cold season pasture of alpine meadow with strong consistency in grassland habitats and vegetation was selected to set up rest-grazing plots in 2018. Five rest-grazing periods were set based on the depth of soil thawing and the coverage of plant re-greening as follows: the beginning of soil surface thawing (RP1), the period when the depth of soil thawing was greater than 10 cm (RP2), the period when the coverage of plant re-greening was 30%~40% (RP3), the period when the coverage of plant re-greening was 70%~80% (RP4), and the period when the height of dominant plants was 5 cm (local traditional rest-grazing period, RP5), which was used as the control. Pseudo-repetitions were used for the study because it is difficult to have large-scale grasslands with the same natural conditions at an altitude of 3000 m (Jing et al., 2022; Jing et al., 2023).

Grazing in cold season pastures usually starts during the withering period of the plant. According to previous observations, grazing during the plant withering and soil frozening periods has a slight effect on grasslands and each treatment is homogeneous. Therefore, grazing started uniformly from March 1 the following year when the soil was frozen. Healthy yaks with consistent coat color and weight (180 ± 20 kg) and Tibetan sheep (45 ± 5 kg) were selected for mixed grazing. Four adult yaks and four Tibetan sheep grazed on RP1, RP2, RP3, and RP3 plots per day, while 16 adult yaks and 16 Tibetan sheep grazed on RP5 plots per day. The area of each plot was calculated on the basis of the livestock carrying capacity as follows:


$$A=\frac{(I-B)\times D\times T}{Y\times U}$$

Where *A* is the plot area (m^2^), *I* is the daily intake by grazing livestock (yak: 5.8 kg of hay, Tibetan sheep: 1.7 kg of hay) (Xu, 2000), *B* is the amount of daily supplementary feeding of grazing livestock (based on a field survey, the daily supplementary feeding of yak and Tibetan sheep was approximately 1.23 kg and 0.22 kg of oat hay, respectively), *D* is the days of grazing (**Table 1**), *T* is the number of grazing livestock, *Y* is the plant yield of the plot (2895 kg·hm^−2^), and *U* is the grassland utilization rate (80%) (Peng et al., 2022).

**Table 1:** Rest-grazing period, grazing time, and area of each plot.

Treatment	Rest-grazing time	Grazing time	Grazing days (d)	Number of grazing livestock	plot area (m^2^)
RP1	March 19~February 28 of the following year	March 1~March 18	18	4 Yaks +4 Tibetan Sheep	1881
RP2	April 2~February 28 of the following year	March 1~April 1	32	4 Yaks +4 Tibetan Sheep	3344
RP3	April 16~February 28 of the following year	March 1~April 15	46	4 Yaks +4 Tibetan Sheep	4807
RP4	May 2~February 28 of the following year	March 1~May 1	62	4 Yaks +4 Tibetan Sheep	6478
RP5	May 21~February 28 of the following year	March 1~May 20	81	16 Yaks +16 Tibetan Sheep	33855

RP1, RP2, RP3, RP4, and RP5 represent the beginning of soil surface thawing, the period when the depth of the soil thawing was greater than 10 cm, the period when the plant re-greening coverage was 30%~40%, the period when the plant re-greening coverage was 70%~80%, and the period when height of dominant plants was 5 cm, respectively.

The time to thaw soils and re-greening plants varies annually due to the influence of interannual climate change. Therefore, the number of grazing livestock was adjusted based on plant yield during the study period.

### 2.3 Plant growth measurements

Five representative quadrats (1 m ×1 m) were selected from each plot in mid-August 2022. The coverage of each species was measured using the needle-punch method. Ten plants of each species were randomly selected and their natural heights were measured. The average value of the measurements was calculated as the height of the plant. The aboveground parts of the plants in the quadrats were cut and placed in envelopes. The harvested plant parts were then dried in an oven at 65°C to a constant weight, and the dry weight was measured to determine the aboveground biomass. A sampling circle was randomly thrown 50 times in each plot and the frequency of each species was recorded. Plants with an importance value greater than 1% were selected to study species niche and interspecific relationships (Li et al., 2024).

### 2.4 Date calculation and analysis

#### 2.4.1 Importance value

Species importance values were calculated using coverage, height, frequency, and aboveground biomass according to the following formula:


$$IV=\frac{\left(RC+RH+RF+RB\right)}{4}$$

Where *IV* is the species importance value, *RC* is the relative coverage, *RH* is the relative height, *RF* is the relative frequency and *RB* is the relative aboveground biomass.

#### 2.4.2 Niche width and niche overlap

Niche width and niche overlap were calculated using Levins (Levins, 1968) and Pianka (Pianka, 1973) indices, respectively, as follows:


$$B_{i}=\frac{1}{\sum_{j=1}^{r}{\left(P_{ij}\right)}^{2}}$$

$$O_{ik}=\frac{\sum_{j=1}^{r}P_{ij}P_{kj}}{\sqrt{\sum_{j=1}^{r}P_{ij}^{2}\sum_{j=1}^{r}P_{kj}^{2}}}$$

Where *B_i_* is the niche width of species *i*, *O_ik_* is the niche overlap of species *i* and *k*. *j* is the quadrat, and *r* is the total number of quadrats. *P_ij_* and *P_kj_* represent the ratio of the important values of species *i* and *k* in quadrat *j* to the sum of the important values of the species in all quadrats, respectively.

#### 2.4.3 Overall association and interspecific correlation

Variance ratio (VR) (Schluter, 1984) and test statistics (W) (Zhang, 2014) were used to analyze the overall association of plant communities as follows:


$$\delta_{T}^{2}=\sum_{i=1}^{S}\frac{n_{i}}{N}(1-\frac{n_{i}}{N})$$

$$S_{T}^{2}=(1/N)\sum_{i=1}^{N}\frac{n_{i}}{N}{\left(T_{j}-t\right)}^{2}$$

$$VR=\frac{S_{T}^{2}}{\delta_{T}^{2}}$$

W=VR×r

Where $\delta_{T}^{2}$ is the variance of occurrence frequency of all species, $S_{T}^{2}$ is the variance of the number of species in all quadrats, *n_i_* is the number of quadrats in which species *i* appears, *N* is the total number of quadrats, *S* is the total number of species, *T_j_* is the number of species appearing in quadrat *j*, and *t* is the average number of species in all quadrats.

Values of VR > 1, VR< 1, and VR = 1 indicates an overall positive, overall negative, and no associations, respectively. The statistic W was used to test the significance of the VR deviation from 1. A W< χ_0.95_^2^(N) or W > χ_0.95_^2^(N) indicated that the overall association was significant, whereas χ_0.95_^2^(N)< W< χ_0.05_^2^(N) indicated that the overall association was not significant.

Spearman rank correlation coefficient (Greig-Smith, 1983) was used to determine interspecific correlations as follows:


$$r\left(i,k\right)=1-\frac{6\sum_{j=1}^{N}(x_{ij}-x_{kj}{)}^{2}}{N^{3}-N}$$

Where *r*(*i*, *k*) is the Spearman rank correlation coefficient of species *i* and *k* in quadrat *j*, *N* is the total number of quadrats, and *x_ij_*, and *x_kj_* are the ranks of species *i* and *k* in quadrat *j*, respectively.

#### 2.4.4 Plant community stability

The stability of plant communities was determined using a modified version of Godron stability method as previously described by Zheng (2000) and Luo et al. (2017). Briefly, the coverage by different plant species in the quadrats was first arranged in a descending order and converted into relative coverage, then gradually accumulated in descending order of relative coverage. Subsequently, the total number of plant species in each quadrat was reciprocated and gradually accumulated based on the order of plant species. Finally, the reciprocal percentage of plant species corresponded to the cumulative relative coverage one by one, and a scatter plot was drawn and fitted with a smooth curve of y = ax^3^+bx^2^+cx+d. The curve intersected with the straight line y = 100-x and the intersection point was the reference point for the stability of the plant community. The Euclidean distance between the intersection coordinates and the stable point coordinates (20, 80) was calculated to quantify the stability of the community.

Data were sorted and analyzed using MS Excel 2021 (Microsoft Corp., Redmond, WA, USA). Niche of species and interspecific relationships were analyzed using the spaa package in R 4.1.2, and mapping was performed using MS PowerPoint 2021 (Microsoft Corp., Redmond, WA, USA) and Hiplot (https://hiplot.org).

## 3 Results

### 3.1 Niche width and niche overlap

Niche width at different rest-grazing periods varied among species (**Table 2**). Niche width of *H. tibeticum* varied substantially among the five rest-grazing periods, with niche width of *C. capillifolia* being larger than that of other species in all treatments. Niche widths of *Elymus nutans*, *M. ruthenia* var. i*nschanicus*, *C. alatauensis*, *Polygonum macrophyllum*, *Anaphalis lacteal*, *Stellera chamaejasme*, and *Thalictrum alpinum* were the largest during RP1. Niche widths of *H. tibeticum*, *Festuca ovina*, *C. alatauensis*, *A. smithii*, *P. macrophyllum*, *Gentiana straminea*, and *T. alpinum* were the smallest during RP5. The overall niche width of plants during RP4 was larger, but smaller during RP1 and RP5.

**Table 2:** Niche width of plants in different rest-grazing periods.

Number	Species	RP1	RP2	RP3	RP4	RP5
1	*Helictotrichon tibeticum*	4.82	4.79	4.86	4.90	1.00
2	*Helictotrichon schellianum*	3.35	4.88	4.86	4.84	4.32
3	*Festuca ovina*	3.92	3.84	3.87	4.69	3.24
4	*Elymus nutans*	4.31	2.99	2.86	4.27	4.09
5	*Medicago ruthenia* var. *inschanicus*	4.82	4.80	4.55	4.13	4.77
6	*Carex capillifolia*	4.94	4.70	4.94	4.88	4.95
7	*Carex alatauensis*	4.70	4.53	4.60	4.47	4.30
8	*Artemisia smithii*	4.76	4.74	4.81	4.82	4.58
9	*Polygonum macrophyllum*	4.96	4.95	4.91	4.92	4.68
10	*Polygonum viviparum*	4.43	4.86	4.82	4.89	4.77
11	*Potentilla discolor*	3.70	2.81	4.22	4.37	2.84
12	*Anaphalis lactea*	4.91	4.46	3.41	4.78	4.72
13	*Allium cyaneum*	4.61	4.69	4.91	4.99	4.79
14	*Saussurea hieracioides*	3.65	4.69	4.60	4.49	4.28
15	*Gentiana straminea*	4.37	4.84	4.47	4.55	4.22
16	*Stellera chamaejasme*	4.98	4.62	3.70	4.96	4.62
17	*Thalictrum alpinum*	4.90	4.43	4.81	4.78	4.28
18	*Oxytropis ochrocephala*	2.10	4.84	2.86	4.59	3.29

RP1, RP2, RP3, RP4, and RP5 represent the beginning of soil surface thawing, the period when the depth of the soil thawing was greater than 10 cm, the period when the plant re-greening coverage was 30%~40%, the period when the plant re-greening coverage was 70%~80%, and the period when height of dominant plants was 5 cm, respectively.

**Figure 1:**
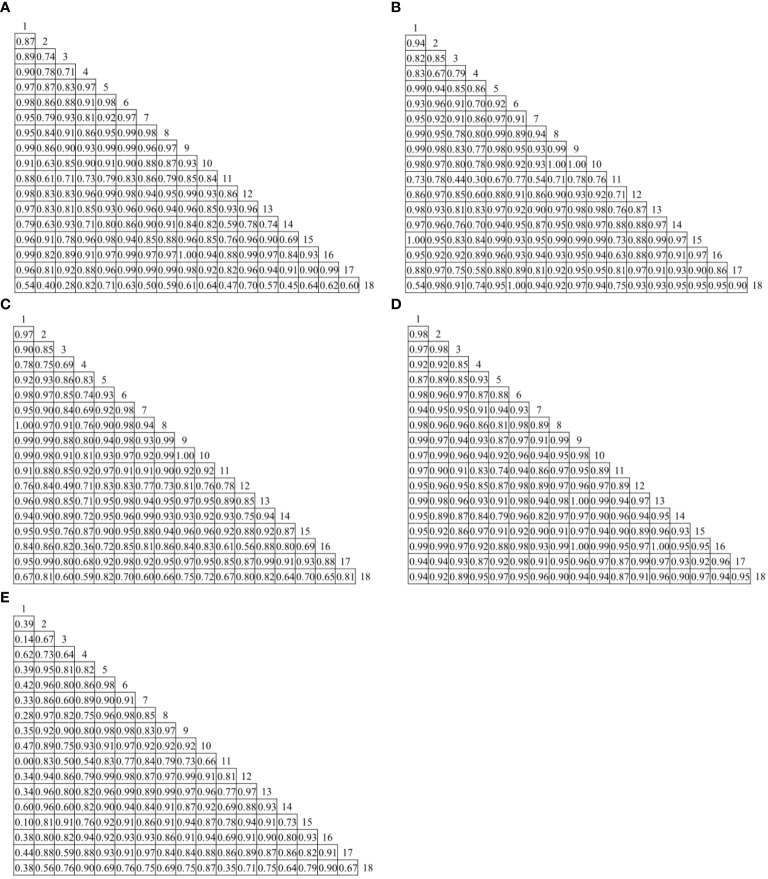
Niche overlap of plants at different rest-grazing periods. **(A–E)** represent at the beginning of soil surface thawing, the period when depth of soil thawing was greater than 10 cm, the period when coverage of plant re-greening was 30%~40%, the period when coverage of plant re-greening was 70%~80%, and the period when height of dominant plants was 5 cm, respectively. 1, *Helictotrichon tibeticum*. 2, *Helictotrichon schellianum*. 3, *Festuca ovina*. 4, *Elymus nutans*. 5, *Medicago ruthenia* var. *inschanicus*. 6, *Carex capillifolia*. 7, *Carex alatauensis*. 8, *Artemisia smithii*. 9, *Polygonum macrophyllum*. 10, *Polygonum viviparum*. 11, *Potentilla discolor*. 12, *Anaphalis lactea*. 13, *Allium cyaneum*. 14, *Saussurea hieracioides*. 15, *Gentiana straminea*. 16, *Stellera chamaejasme*. 17, *Thalictrum alpinum*. 18, *Oxytropis ochrocephala*.

Niche overlap of plant species during RP1, RP2, RP3, and RP4 was 97% above 0.50, while the number of plant species with niche overlap below 0.50 during RP5 was 15, accounting for 9.80% of the total number of plants (**Figure 1**). The species pairs with the largest niche overlap during RP1, RP2, RP3, RP4, and RP5 were *P. macrophyllum* and *S. chamaejasme* (0.995), *A. smithii* and*P. viviparum* (0.996), *H. tibeticum* and *A. smithii* (0.998), *Allium cyaneum* and*S. chamaejasme* (0.997), *M. ruthenia* var. *inschanicus* and *A. lacteal* (0.994), respectively. The niche overlap of *H. tibeticum* and *Potentilla discolor* was 0 during RP5. The overall niche overlap of plants during RP4 was larger, but that during RP1 and RP5 was smaller.

### 3.2 Overall association and interspecific correlation

The VR of RP1, RP2, and RP5 was greater than 1 and an overall positive interspecific association was observed. The VR of RP3 and RP4 was less than 1 and an overall negative interspecific association was observed (**Table 3**). According to the χ^2^ test critical value table, χ_0.95_^2^(5) and χ_0.05_^2^(5) were 1.145 and 11.071, respectively. The W of RP1, RP3, RP4, and RP5 were in the interval (1.145, 11.071) and the interspecific associations were not significant. The W of RP2 was not in the interval (1.145, 11.071) and the interspecific associations were significant.

**Table 3:** Overall association of plants at different rest-grazing periods.

Treatment	*δ_T_^2^*	*S_T_^2^*	*VR*	*W*	χ^2^ threshold (χ^2^0.95(5), χ^2^0.05(5))	Result
RP1	0.96	1.60	1.67	8.33	(1.145, 11.071)	No significant positive association
RP2	1.36	3.20	2.35	11.76	(1.145, 11.071)	Significant positive association
RP3	1.12	1.04	0.93	4.64	(1.145, 11.071)	No significant negative association
RP4	0.40	0.24	0.60	3.00	(1.145, 11.071)	No significant negative association
RP5	1.28	1.84	1.44	7.19	(1.145, 11.071)	No significant positive association

RP1, RP2, RP3, RP4, and RP5 represent the beginning of soil surface thawing, the period when the depth of the soil thawing was greater than 10 cm, the period when the plant re-greening coverage was 30%~40%, the period when the plant re-greening coverage was 70%~80%, and the period when height of dominant plants was 5 cm, respectively.

**Figure 2:**
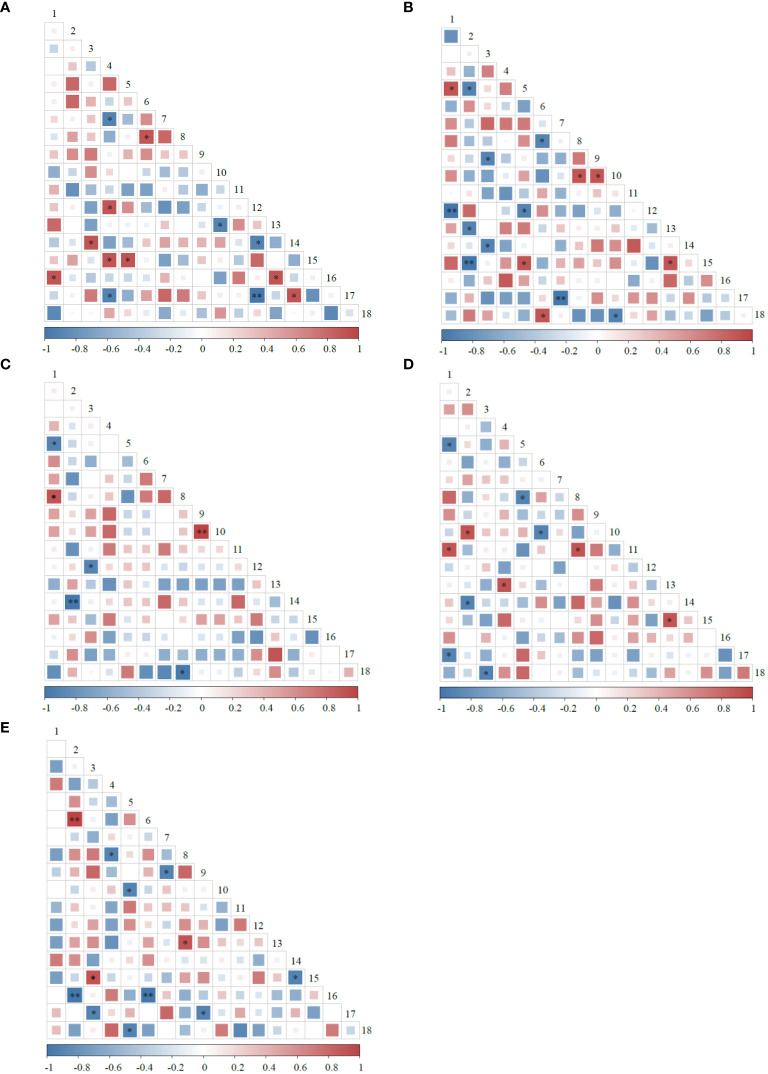
Semi-matrix diagram of the Spearman rank correlation coefficient test for plants at different rest-grazing periods. **(A–E)** represent at the beginning of soil surface thawing, the period when depth of soil thawing was greater than 10 cm, the period when coverage of plant re-greening was 30%~40%, the period when coverage of plant re-greening was 70%~80%, and the period when height of dominant plants was 5 cm, respectively. * and ** indicate significant correlations at the 0.05 and 0.01 levels, respectively. 1, *Helictotrichon tibeticum*. 2, *Helictotrichon schellianum*. 3, *Festuca ovina*. 4, *Elymus nutans*. 5, *Medicago ruthenia* var. *inschanicus*. 6, *Carex capillifolia*. 7, *Carex alatauensis*. 8, *Artemisia smithii*. 9, *Polygonum macrophyllum*. 10, *Polygonum viviparum*. 11, *Potentilla discolor*. 12, *Anaphalis lactea*. 13, *Allium cyaneum*. 14, *Saussurea hieracioides*. 15, *Gentiana straminea*. 16, *Stellera chamaejasme*. 17, *Thalictrum alpinum*. 18, *Oxytropis ochrocephala*.

Among the 153 species pairs composed of 18 plants at different rest-grazing periods, negatively correlated species pairs were more than positively correlated species pairs (**Figure 2**). Deferred rest-grazing period decreased correlated species pairs. The significantly positively correlated species pairs during RP1 were more than the significantly negatively correlated species pairs (*P<* 0.05). In addition, the significantly positively correlated species pairs during RP2, RP3, RP4, and RP5 were less than the significantly negatively correlated species pairs (*P<* 0.05). A significant positive correlation was observed between *M. ruthenia* var. *inschanicus* and *G. straminea* during RP1 and RP2, while a significant negative correlation was observed between *Helictotrichon schellianum* and *Saussurea hieracioides* during RP3 and RP4 (*P<* 0.05). Significantly positively correlated species pairs (*P. macrophyllum* and *P. viviparum*, and *H. schellianum* and *C. capillifolia*) were observed during RP3 and RP5, respectively (*P<* 0.01).

### 3.3 Stability of the plant community

**Figure 3:**
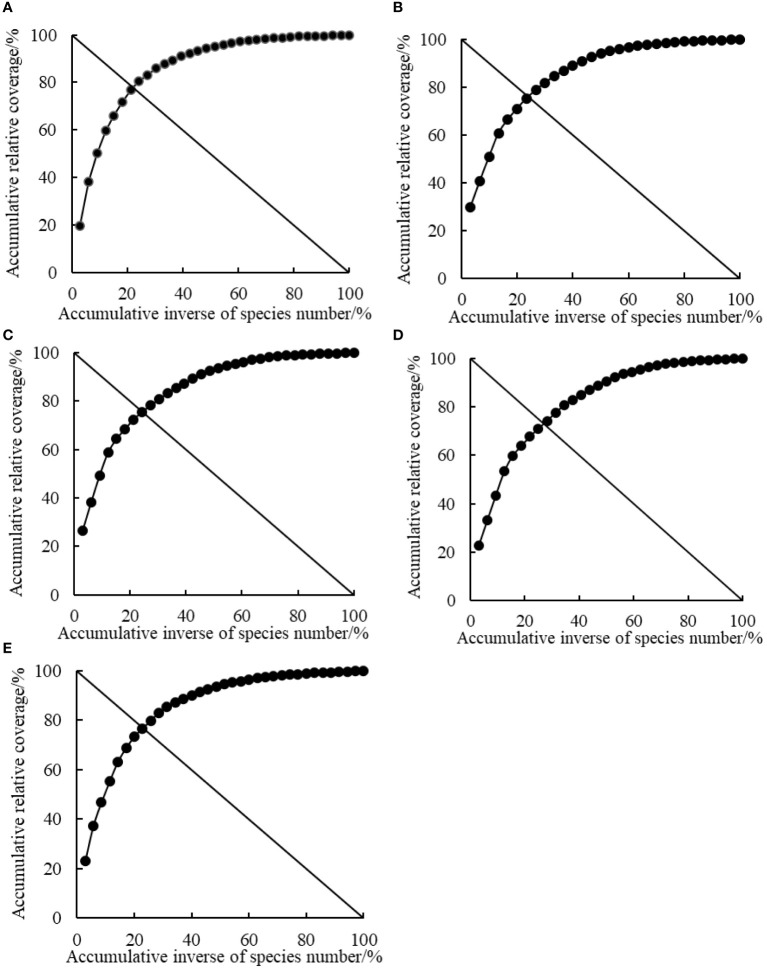
Simulated curves of plant community stability at different rest-grazing periods based on Godron stability analysis. **(A–E)** represent the beginning of soil surface thawing, the period when depth of soil thawing was greater than 10 cm, the period when coverage of plant re-greening was 30%~40%, the period when coverage of plant re-greening was 70%~80%, and the period when height of dominant plants was 5 cm, respectively.

A short Euclidean distance between the intersection of coordinates and stable reference points (20, 80) of the plant community indicated a more stable plant community, whereas a long Euclidean distance indicated an unstable plant community. According to the results, the stability of the plant communities at different rest-grazing periods was in the order of RP5 > RP1 > RP2 > RP3 > RP4 (**Table 4** and **Figure 3**).

**Table 4:** Stability of plant communities at different rest-grazing periods based on Godron stability analysis.

Treatment	Fitting curve	R^2^	Intersection coordinates	Euclidean distance
RP1	y = 0.0002x^3^ - 0.0422x^2^ + 2.9748x + 28.143	0.9886	(23.36,77.43)	4.23
RP2	y = 0.0002x^3^ - 0.0409x^2^ + 3.0016x + 23.904	0.9939	(23.36,76.17)	5.09
RP3	y = 0.0002x^3^ - 0.0396x^2^ + 2.9307x + 24.169	0.9884	(24.04,75.97)	5.71
RP4	y = 0.0002x^3^ - 0.0368x^2^ + 2.9039x + 18.589	0.9930	(26.69,72.88)	9.77
RP5	y = 0.0002x^3^ - 0.0490x^2^ + 3.3648x + 20.770	0.9881	(22.86,77.02)	4.13

## 4 Discussion

### 4.1 Effects of different rest-grazing periods on niche

Mutual adaptation between individuals and the environment is achieved primarily through three processes: niche selection, niche compliance, and niche construction (Müller et al., 2020). Niche theory plays an important role in the study of plant populations. The niche width of *C. capillifolia* was larger at different rest-grazing periods. This could be because the roots of *C. capillifolia* were interwoven to form a mat-shape layer on the soil surface, which has a strong resistance to grazing livestock (Miehe et al., 2019), thus improving its survival in the plant community. Niche widths of *E. nutans*, *M. ruthenia* var. i*nschanicus*, *C. alatauensis*, *P. macrophyllum*, *A. lacteal*, *S. chamaejasme*, and *T. alpinum* were the largest during RP1. The results suggest that the species during RP1 have high resource utilization efficiency and environmental adaptability, which contribute to their wide distribution range, and maintain the stability of internal and external environmental factors that act on the plant community (Zhang et al., 2018). The observation could be attributed to the fact that RP1 was beneficial for early plant re-greening, making it easier for plants to seize the biological space and gain competitive advantage (Rathcke and Lacey, 1985), especially during the limited growth season in high-altitude areas (Wang et al., 2014; Shen et al., 2015; Li et al., 2022). The smallest niche widths were observed for *H. tibeticum*, *F. ovina*, *C. alatauensis*, *A. smithii*, *P. macrophyllum*, *G. straminea*, and *T. alpinum* during RP5, indicating that these species during RP5 have low adaptability to environmental changes and few suitable habitats, and may tend to specialize (An et al., 2021). The overall niche width during RP4 was larger than that observed during other rest-grazing periods because delayed rest-grazing reduces the soil nutrients returned by plants (Jing et al., 2022), in turn, decreasing the resources available for plant use. Plants generally increase their niche widths and improve their competitive ability to obtain the resources necessary for growth (Zhao et al., 2023).

Studies have shown that populations with relatively large niche width have a greater chance of niche overlap with other populations, and vice versa (Xu et al., 2007). This study found that the maximum niche overlap during each rest-grazing period occurred between two species with larger niche width, which is consistent with the results of Zhao et al. (2023) showing that niche width of plant species was positively correlated with niche overlap. Niche overlap of *H. tibeticum* and *P. discolor* was 0 during RP5, indicating low similarity of their resource utilization. This may be because in order to adapt to grazing stress, plants usually reduce their niche width to adapt to the environment, consequently altering the competitive exclusion of populations, which in turn affects the niche overlap between species (Yin et al., 2023). The overall niche overlap of plants during RP4 was laeger than that observed during the other rest-grazing periods. The observation could be attributed to the grazing avoidance strategy of plants; that is increasing niche overlap to reduce feeding and trampling by livestock, thus enhancing the survival of species (Lv et al., 2014). Niche overlap of plants during RP5 was samller than that observed during the other rest-grazing periods, which could be attributed to the growth and development of plant species that are threatened by livestock feeding and trampling, and subsequently require more resources to maintain their growth. Therefore, competition between plant species for spatial resources is enhanced, in turn, resulting in niche separation and a considerable reduction in niche overlap (Lv et al., 2014). Moreover, interference from livestock feeding, trampling, and other behaviors reduces the competitive ability of some plant species to obtain resources. To consolidate their intensity of aggregation increases, which decreases the niche overlap between species (Wang et al., 2022).

### 4.2 Effects of different rest-grazing periods on interspecific relationships

Promoting and interfering effects (i.e., positive and negative correlation effects) occur among species during community succession, and the balance between these effects is associated with the biological characteristics of species, environmental conditions, as well as spatial and temporal distribution of species (Aguiar and Sala, 1994; Callaway et al., 2002). In this study, positive associations were observed among plant species during RP1 and RP2, indicating that earlier rest-grazing can weaken interspecific competition for environmental resources among these species and result in synergistic interspecific relationships. Negative associations were observed among plant species during RP3 and RP4, while a positive association was observed among plant species during RP5. The overall association changed from a negative association to a positive association, which is consistent with the results of Wang et al. (2021) showing that light grazing showed a negative association, while moderate and heavy grazing showed a positive association in Stipa breviflora desert steppe. The reason is the limiting factors for plant re-greening were primarily related to water, light, soil nutrients, and other resources. Owing to variations in the ability of plant populations in a community to utilize resources, plants generally show a competitive relationship (Wang et al., 2021). Furthermore, the cyperaceae plants are the dominant species in alpine meadows, which regreen in early spring and complete their reproductive life cycle (Jing et al., 2023). Individual plant species dominate the plant community and exert inhibitory effects on the growth of other plant species (Wu et al., 2022). Feeding and trampling by livestock were the main factors limiting the growth of the plant population during RP5, which limited the availability of resources. To maintain their position and role in the community, plant populations form long-term synergistic effects as an evolutionary strategy to deter grazing, resulting in an overall positive association, showing an affinity relationship (Liu et al., 2019).

According to our results, the negatively correlated species pairs were more than the positively correlated species pairs during different rest-grazing periods, suggesting that the adaptability of the predominant plant species to the environment varied, with niche overlap and competition for water, light, soil nutrients, and other resources (Liu et al., 2019). Deferred rest-grazing period decreased correlated species pairs, indicating a reduction in interspecific relationships between plants (Zhao et al., 2021). Significantly positively correlated species pairs were more than the significantly negatively correlated species pairs during RP1, suggesting that the plant community developed toward a stable direction and finally achieved stable coexistence among species (Liu et al., 2010). A significant positive correlation was observed between *M. ruthenia* var. *inschanicus* and *G. straminea* during RP1 and RP2, suggesting that the ecological characteristics of both species are complementary and can promote their growth (Zhao et al., 2021). A significant negative correlation was observed between *H. schellianum* and *S. hieracioides* during RP3 and RP4, suggesting strong interspecific competition and interference between the two plant species, which is associated with livestock feeding and trampling (Zhao et al., 2021).

### 4.3 Effects of different rest-grazing periods on plant community stability

Stability of the plant community can reflect variations in plant responses to biotic or abiotic factors (van Moorsel et al., 2021). Studies have shown that external environmental disturbances and interspecific relationships impact the stability of alpine meadow communities (Luo et al., 2017). In this study, an overall positive association was observed among the evaluated plant species and the stability of the plant communities was high during RP1, RP2 and RP5. However, an overall negative association was observed among plant species and the stability of plant communities was low during RP3 and RP4. The results suggest that initiating rest-grazing during soil thawing is ideal for the succession of plant communities in alpine meadows toward a more stable plant community (Shen et al., 2022). However, initiating rest-grazing during plant re-greening results in relatively unstable plant communities. The observation could be because rest-grazing during soil thawing accelerates nutrient cycling (Jing et al., 2022) and promotes redistribution of resources, which is conducive to maintaining ecosystem stability (Wang et al., 2019b). However, grazing during soil thawing caused plants are in a low or equal compensation state when re-greening, which leads to low plant community stability (Gao et al., 2021). Grazing for a long period during RP5 affected plants, leading to the development of corresponding adaptive mechanisms by plant communities, which in turn resulted in high plant community stability.

This study describes the effects of rest-grazing period on species niche, interspecific relationship and stability of plant communities in alpine meadow, and results can provide a reference for plant community competition mechanism and grazing management of alpine grassland. We can further research the mechanisms underpinning these the responses and the potential wider impacts on ecosystem structure and function.

## 5 Conclusion

The niche width at different rest-grazing periods varied among plant species. The largest niche overlap was observed between two species with large niche width. Small niche width and niche overlap of plant species, overall positive association, and high community stability were observed during soil thawing and local traditional rest-grazing periods. On the contrary, large niche width and niche overlap of plant species, overall negative association, and low community stability were observed during the plant re-greening period. Deferred rest-grazing period decreased correlated species pairs. Thus, rest-grazing in alpine meadows should begin as soon as possible to promote healthy and sustainable utilization of grasslands.

## Data availability statement

The original contributions presented in the study are included in the article/supplementary material. Further inquiries can be directed to the corresponding author.

## Ethics statement

Ethical approval was not required for the studies involving humans because the manuscript does not address issues that require ethical approval. The studies were conducted in accordance with the local legislation and institutional requirements. The participants provided their written informed consent to participate in this study.

## Author contributions

KM: Investigation, Methodology, Writing – original draft, Writing – review & editing. QH: Methodology, Writing – review & editing. CX: Investigation, Writing – review & editing. YC: Investigation, Writing – review & editing. HY: Methodology, Writing – review & editing. JX: Methodology, Writing – review & editing. XY: Funding acquisition, Project administration, Supervision, Writing – review & editing.
